# Histone modifications change with age, dietary restriction and rapamycin treatment in mouse brain

**DOI:** 10.18632/oncotarget.4137

**Published:** 2015-05-20

**Authors:** Huan Gong, Hong Qian, Robin Ertl, Clinton M. Astle, Gang G. Wang, David E. Harrison, Xiangru Xu

**Affiliations:** ^1^ Department of Anesthesiology, Yale University School of Medicine, New Haven, CT, USA; ^2^ The Jackson Laboratory, Bar Harbor, ME, USA; ^3^ Department of Biochemistry and Biophysics and Lineberger Comprehensive Cancer Center, University of North Carolina at Chapel Hill, Chapel Hill, NC, USA; ^4^ Max Planck Institute for Biology of Ageing, Cologne, Germany; ^5^ The Key Laboratory of Geriatrics, Beijing Institute of Geriatrics, Beijing Hospital, Ministry of Health, Beijing, China; ^6^ Center for Natural and Health Sciences, Marywood University, Scranton, PA, USA

**Keywords:** gerotarget, gerontology, health span, mTOR, rapalogs

## Abstract

The risk of developing neurodegenerative disorders such as Alzheimer's disease (AD) increases dramatically with age. Understanding the underlying mechanisms of brain aging is crucial for developing preventative and/or therapeutic approaches for age-associated neurological diseases. Recently, it has been suggested that epigenetic factors, such as histone modifications, maybe be involved in brain aging and age-related neurodegenerations. In this study, we investigated 14 histone modifications in brains of a cohort of young (3 months), old (22 months), and old age-matched dietary restricted (DR) and rapamycin treated BALB/c mice. Results showed that 7 out of all measured histone markers were changed drastically with age. Intriguingly, histone methylations in brain tissues, including H3K27me3, H3R2me2, H3K79me3 and H4K20me2 tend to disappear with age but can be partially restored by both DR and rapamycin treatment. However, both DR and rapamycin treatment also have a significant impact on several other histone modifications such as H3K27ac, H4K16ac, H4R3me2, and H3K56ac, which do not change as animal ages. This study provides the first evidence that a broad spectrum of histone modifications may be involved in brain aging. Besides, this study suggests that both DR and rapamycin may slow aging process in mouse brain via these underlying epigenetic mechanisms.

## INTRODUCTION

In industrialized countries, life expectancy is rapidly rising, and this has led to an increasing incidence of age-related neurodegenerative disorders including Alzheimer's disease (AD), Parkinson disease (PD) and Huntington Disease (HD). The lack of effective therapies that can halt and/or reverse the progression of these disorders creates an enormous burden on both affected individuals and society as a whole. Developing preventative or therapeutic interventions for such conditions demands deeper understanding of the processes underlying normal brain aging [1-2]. Brain aging is marked by a gradual decline in cognitive function, which is often correlated with age-dependent deterioration of synaptic function in brain regions crucial for memory formation and consolidation, such as the hippocampus and prefrontal cortex [3-4]. The neurobiological processes underlying these age-related learning and memory deficits include aberrant changes in gene transcription that eventually affects the plasticity of the aged brain. Changes in gene expression in neurons were thought to take place during brain aging, and analysis of regions of the hippocampus and front cortex by microarray has confirmed this [5-7]. The molecular mechanisms underlying these changes in gene expression and the regulation are largely unknown, but recent studies point to a novel possibility that the dysregulation of epigenetic control of gene expression may be involved [8-10].

The term “epigenetics” was first coined by Conrad Waddington in the 1940s to describe interactions of genes with their environment during development stages [11]. The first suggestion that DNA methylation might play an important biological role was made by Griffith and Mahler in 1969 indicating that it could provide a basis for long term memory in the brain [12]. The two most widely studied epigenetic codes are DNA methylation and histone modifications. Epigenetic regulation is a key transcriptional mechanism that alters gene expression by altering the structure and/or conformation of chromatin but does not change the basic genetic code. Many fundamental cellular processes are affected by epigenetic modulation, and it has become evident that chromatin-based epigenetic mechanisms underlie important aspects of the aging process. Recent reports indicate that chromatin remodeling via histone acetylation plays a crucial role in regulating synaptic and cognitive function in aging and neurodegenerative brains [13-16]. Increasing histone acetylation by inhibition of histone deacetylase (HDAC) enhances gene transcription and improves hippocampal long-term potentiation (LTP) or memory functions in several experimental models of neurological diseases [17-19], indicating that dysregulation of chromatin acetylation may be involved in certain forms of cognitive impairment [20-21]. Histone methylation can occur on either lysine (K) or arginine (R) residues, and can be mono-, di- or trimethylated, and either activation or repression is dependent upon the particular histone residue, that is modified [22-23]. Therefore, identifying new histone modifications that may be involved in brain aging has a potential to provide critical insights into the neurobiology of aging and possibly the etiology of neurodegenerative disorders.

Dietary restriction (DR) delays aging and improves resistance to disease in a fashion that is evolutionarily conserved from yeast to primates and humans [24-25], though controversy was stirred lately on an NIH-initiated primate cohort study [26]. These beneficial effects include, in mammals, the attenuation of age-associated cognitive impairment and neurodegeneration [27-28]. More specifically, synaptic plasticity was shown to be enhanced by DR, as evidenced by increased LTP [29]. rapamycin, an antibiotic, is already in use for suppressing the immune system in transplant patients and has been used in the treatment of certain cancers for decades. Lately rapamycin treatment produced remarkable effect of extending lifespan even though it was not started until the mice had lived 600 days - equivalent to human being aged 60 years [30]. A similar benefit on lifespan has also been observed when started on the drug at a younger age (9 months old) [31]. Blagosklonny discussed the link between aging and diseases by rapamycin treatment [32]. Moreover, Rapamycin has been found to have the potential for reversing some of the effects of premature aging [33]. Interestingly, rapamycin treatment suppresses brain aging in senescence-accelerated OXYS rats [34] and results in an improvement in cognitive functions that normally decline with age in mice [35-36]. Furthermore, inhibition of mTOR by rapamycin can slow or block AD progression in a transgenic mouse model of the disease [37].

Finally, epigenetic factors are not only inheritable but also reversible. Thus, these factors, such as histone acetylation and methylation, may participate in the modulation of cell plasticity in responses to environmental cues in the brain. It is of great interest to hypothesize that intervention approaches such as DR and rapamycin administration, which not only extending the lifespan of animals under certain conditions but also promote certain brain functions in mice, may impact some age-induced post-translational modifications of histones. In the present study, we aim to test this hypothesis by examining expression of a panel of 14 histone modifications in brains of a mouse cohort in which the mice were maintained for over 2 years either eating ad libitum, or with either DR or rapamycin treatment.

## RESULTS

To identify the histone modifications which either change with age and/or respond to age-interventions including DR and rapamycin treatment in mouse brain, a cohort of BALB/c mice with groups of young (3 months), old (22 months), old age-matched DR, and old age-matched rapamycin treatment and a panel of 14 histone antibodies against specific methylations or acetylations in residues of both lysine (K) and arginine (R) were applied in this study (Table 1). Surprisingly, over 50% of detected histone modifications in brains from these mice were altered as a result of the age of animals or the age-interventions. Interestingly, those modifications have not been reported to be associated with brain aging or age-interventions until now.

**Table 1:** Summarization of measured histones in BALB/c brains

Antibodies\experiment	young vs old	RAL vs old	DR vs old
H3R2me2	↘	↗	↗
H3K27me3	↘	↗	↗
H3K79me3	↘	↗	↗
H4K20me2	↗	↘	→
H3K4me	↘	↘	↘
H3K4me2	↗	↗	↗
H3K18ac	↗	↗	↗
H4K16ac	→	↘	→
H3K27ac	→	↗	↗
H3K56ac	→	→	↘
H4R3me2	→	→	↗
H4K20me	→	→	→
H3K4me3	→	→	→
H3K9me3	→	→	→

*↘ reduced expression ↗ increased expression → no change

**Figure 1:**
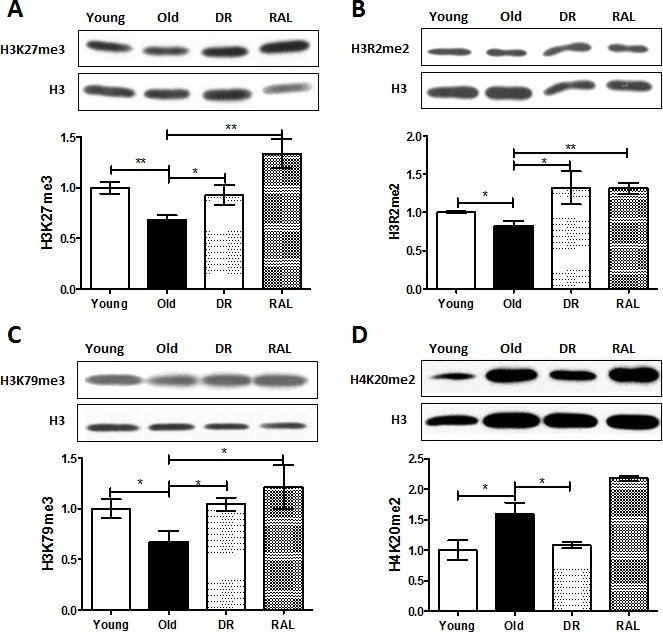
Histone methylations are changed with age but the age-associated change is prevented by DR and/or rapamycin treatment in mouse brain Representative western blots (upper) and quantitative densitometric analysis (lower) of H3K27me3 **A.**, H3R2me2 **B.**, H3K79me3 **C.** and H4K20me2 **D.** in brains from 3 months old ad libitum (Young), 22 months old ad libitum (Old), 22 months old dietary restricted (DR) and 22 months old rapamycin treatment (RAL) BALB/c mice. Histone modification levels were normalized to total H3 content. The values (mean±S.E.M.) are expressed as a percentage relative to the 3 months old group. *n* = 3~5 animals per goup. (**p < 0.01, *p < 0.05 Old versus Young, DR versus Old, RAL versus Old).

Among the measured histone modifications, the levels of H3K27me3, H3R2me2 and H3K79me3 in brains of old (22 months) mice were reduced to 68% (*p* < 0.01), 83% (*p* < 0.05) and 67% (*p* < 0.05) respectively when compared to 3-month old animals. However, both DR and rapamycin treatment prevented the age-induced losses of H3K27me3, H3R2me2 and H3K79me3. Specifically, DR restored the levels of these histones of old age group to 92.5%, 132% and 104% of young age group, respectively, and likewise, rapamycin exhibited an even stronger impact by increasing the levels of these histone methylations to 133%, 132% and 120% of those seen in the young age group (Figure 1A-1C). Interestingly, the level of H4K20me2 is increased with age (159%, *p* < 0.05) when in mice on DR, this was reduced to 108%. Rapamycin treatment, contrary to DR, was unable to suppress the age-induced H4K20me2 expression (Figure 1D).

**Figure 2:**
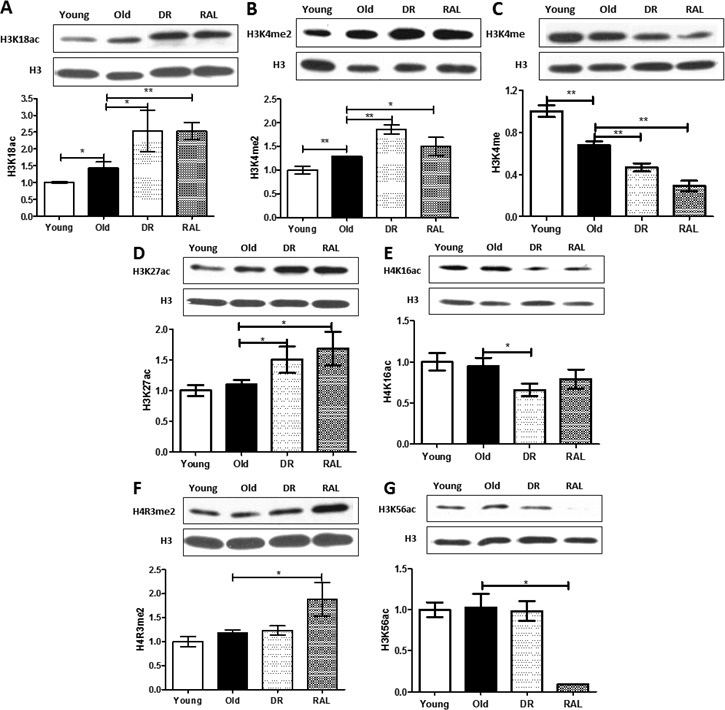
Distinctive impact of DR and/or rapamycin treatment on histone modifications in mouse brain Representative western blots (upper) and quantitative densitometric analysis (lower) of H3K18ac **A.**, H3K4me2 **B.**, H3K4me **C.**, H3K27ac **D.**, H4K16ac **E.**, H4R3me2 **F.** and H3K56ac **G.** in brains from 3 months old ad libitum (Young), 22 months old ad libitum (Old), 22 months old dietary restricted (DR) and 22 months old rapamycin treatment (RAL) BALB/c mice. Histone modification levels were normalized to total H3 content. The values (mean±S.E.M.) are expressed as a percentage relative to the 3 months old group. *n* = 3~5 animals per goup. (**p < 0.01, *p < 0.05 Old versus Young, DR versus Old, RAL versus Old).

In addition, levels of H3K18ac, H3K4me2 and H3K4me were also changed with brain aging (Figure 2A-2C). The levels of H3K18ac and H3K4me2 increased 44% (p < 0.05) and 29% (*p* < 0.01), respectively, and the level of H3K4me decreased 32% (p < 0.01) in old brains when compared to that of young brains. The levels of H3K18ac and H3K4me2 were enhanced further by DR (76%, *p < 0.05* and 44%, *p < 0.01,* respectively) and rapamycin (77%, *p < 0.01* and 16%, *p* < 0.05, respectively) when compared to the old group. Furthermore, the level of H3K4me declined with age and was further diminished by both DR and rapamycin (51% and 70%, *p < 0.01,* respectively), when compared with the old group.

Both DR and rapamycin also demonstrated unique functions in modulating the expression of histone modifications that do not respond to age. Four transcriptional activation histone modifications, H3K27ac, H4K16ac, H4R3me2 and H3K56ac, do not shown detectable differences between young and old brains, but their expression was changed significantly by DR and/or rapamycin treatment (Figure 2D-2G). Compared to the age-matched controls, both DR and rapamycin treatment increased the expression of 37% and 54%, respectively of H3K27ac (*p* < 0.05); DR alone decreased expression of H4K16ac to 69% (*p < 0.05*). Rapamycin increased expression of H4R3me2 to 188% but decreased the expression of H3K56ac over 10 times (*p < 0.05*).

## DISCUSSION

The organization of the eukaryotic genome into chromatin enables DNA to fit inside the nucleus. Essentially, histone proteins form a “spool” which allows DNA to be wound around them. Post-translational modification of histones has an effect on the tightness of DNA winding; it thus regulates the the accessibility of proteins such as transcriptional activators/repressors to the DNA to facilitate genomic functions by turning on and off of gene expression. Tight winding keeps genes switched off, while loosening the packaging allows gene to be turned on. Therefore, remodeling of chromatin by histone modifications, such as acetylation, methylation and phosphorylation, is a key mechanism in controlling gene transcription in a variety of important biological processes [38].

Recent work suggests that histone acetylation has a critical role in age-associated declines in cognitive functions. Rodents exhibit a transient increase in histone acetylation after exposure to different learning paradigms [9]. Restoration of the expression of H4K12ac in mouse brain leads to a recovery of cognitive functions [16]. More importantly, rodents, after treatment with histone deacetylase inhibitors (HDACis), have demonstrated that targeting histone acetylation has emerged as a promising strategy for therapeutic intervention in age-related cognitive function decline and neurodegenerative diseases [17, 39].

Histone methylation is another important and widespread type of chromatin modification that is known to influence biological processes in the context of development and cellular responses [21]. Histone methylation can occur on either lysine (K) or arginine (R) residues, and these can be mono-, di- or trimethylated, and either activation or repression is dependent upon the particular lysine residue that is modified [21]. For instance, H3K4me3, an active mark for transcription, is upregulated in hippocampus one hour following contextual fear conditioning [40]. Interestingly, a line of exciting evidence indicates that specific histone methylations, H3K4me3 and H3K27me3, may act as a regulator of lifespan was confirmed by studies in *C. elegans* [41-42]. In addition, studies have shown that the expression of p16INK4a, a cell-cycle regulatory protein and an aging biomarker, can be regulated by H3K27me3 during cellular senescence [43]. However, whether histone methylation is involved in brain aging is still an open question. Encouragingly, we identified alterations in the methylation level of several hisotone residues including H3K27me3, H3R2me2, H3K79me3 and H4K20me2 with aging of mouse brains (Figure 1). The arginine residue methylation of Histone 3, H3R2me2, has never been associated with any brain function previously. More interestingly, these age-associated alterations are prevented by DR and/or rapamycin treatment (Figure 1). However, further work needs to be done to (1) study the functional significance of these histone changes in mouse brain aging, and (2) identify the transcriptional targets of these age-associated histone markers in neuronal cells.

Brain aging and its direct consequences, such as degenerative diseases and even death, are inevitable; however, scientific advances in understanding basic aging mechanisms have made it much more feasible to postpone aging processes and to increase the animal lifespan using some specific regimens including DR and rapamycin administration. DR has been studied for decades in extending lifespans across species but the underlying molecular mechanism is not fully clear yet [24, 27]. Rapamycin, a bacterial product first isolated from soil on Easter Island, is the first drug intervention to reliably increase mammalian lifespan by 10% or more [30-31]. More intriguingly, Kolosova et. al. first described that rapamycin prevents brain aging in rat [34]; rapamycin also produces an improvement in age-associated cognitive functions in mouse[35-37]. We therefore wondered if epigenetic modifications play a role in mediating the beneficial effects of both DR and rapamycin in mouse brain aging. The results from our study unexpectedly exhibited that both DR and rapamycin can restore, at least partially, the age-related alterations in histone methylation levels (Figure 1). This may put forward a novel epigenetic mechanism of beneficial age-interventions. However, it is still not known whether these epigenetic responses have any relevance with the classic DR-driven IGF/Insulin and rapamycin-driven mTOR pathways in the brain. In addition, age and DR or rapamycin exhibit similar effects on the overall level of several histone modifications, such as H3K18ac, H3K4me2 and H3K4me (Figure 2A-2C). It implies that those histone modifications may play dual regulatory roles in mediating both age and age-interventions. One possible favorable explanation is that age-adaptive cellular stress responses could be enhanced by DR and/or rapamycin to strengthen neuronal networks and plasticity [44]. Our study also showed that histone modifications such as H4K16ac and H3K56ac were stable with age but regulated by DR or rapamycin treatment (Figure 2D-2G). Interestingly, H4K16ac has been identified as DR regulated histone marker that may be modulated by Sirt1 regulation [45]. H3K56ac has also been identified biochemically as a direct target of mTOR inhibition by a chemical genomic screening recently [46]. The latter suggests a novel chromatin-regulating role for mTOR signaling.

In summary, this study has examined a panel of histone modifications in mouse brain, and different patterns of histone modifications are responsive to normal aging and age-interventions including DR and rapamycin treatment. Based on these findings, we propose that dysregulation of chromatin remodeling may occur and contribute to brain aging as well as cognitive impairments observed in age-related neurodegenerative diseases such as AD. Promising interventions targeting age-related conditions may prevent the associated change of histone modifications or bypass age-induced cognitive deficit with compensational pathways. We know that a large amount of careful work needs to be carried out in the future uncover the functional significance of these promising histone modifications. We hope this study offers enriched epigenetic information to brain aging and neurodegeneration researchers and encourages the field to consider and understand the role of epigenetic regulation in neuroplasticity and cognitive function.

## MATERIALS AND METHODS

### Animals

**Figure 3:**
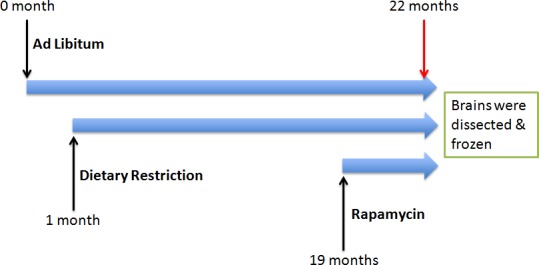
Experimental design A cohort of BALB/C mice were subjected to ad libitum, Dietary restriction and rapamycin at various time points.

BALB/C female mice were produced and maintained at the Jackson Laboratory (Bar Harbor, ME). At weaning (3 weeks of age), they were housed 4 per box in weaning cages. These cages were assigned to one of 3 feeding regimens using Purina LabDiet's 5LG6 irradiated formulation of the NIH-31 (4% fat) diet. Groups of ad libitum (AL) fed mice, both young (3 months) and old (22 months), were given uninhibited access to grain. A Group of DR mice were fed with 70% of the AL feeding rate after weaning and continued lifelong (22 months of age). For group of rapamycin-treated mice, the rapamycin was encapsulated, and 2.24 mg of rapamycin per kg body weight/day was administered (equivalent to AL feeding) for 3 months starting when the animals were 19 months of age [30]. All procedures were carried out as indicated in Figure 3 and approved by the animal care and use committee of the Jackson Laboratory, and staff veterinarians monitored mice on a regular basis, finding no pathogens.

### Tissues preparation

Mice were sacrificed by cervical dislocation according to the approved protocol. Then the health status of mice was first checked anatomically. The whole brains including all major parts of the brain of all mice without liver/kidney problems, severe inflammations' or tumors that could impact brain health (e.g., pituitary tumors) were dissected out and snap frozen in liquid nitrogen, ground into powder with mortar and pestle in liquid nitrogen and then stored at −80°C.

### Acid extraction of histones

Core histone proteins were extracted from the mouse whole brain samples using an acid extraction method as described by Shechter et al. with slight modifications [47]. Briefly, all procedures were performed on ice, and all solutions were chilled to 4°C before use unless otherwise indicated. All centrifugation steps were performed at 4°C. Thirty to sixty microgram whole brain powder was homogenized in ice-cold PBS containing the following: 1mM phenylmethylsulfonyl fluoride, 1x complete protease inhibitor cocktail (Roche, cat. no. 1697498), 5mM sodium butyrate (Sigma-Aldrich, B5887). The homogenates were centrifuged at 1000g for 10min and the supernatant was discarded. The pellet was then resuspended in 1 ml hypotonic lysis buffer (10mM Tris-Cl pH 8.0, 1 mM KCl, 1.5 mM MgCl2, 1 mM DTT), incubated for 30 min on rotator at 4°C, centrifuged at 14,000g for 10 min, and the supernatant (cytoplasmic fraction) was aspirated. The pellet (nuclear fraction) was then resuspended in 0.5 ml of 0.4N H2SO4, incubated for 30 min to overnight and centrifuged at 16,000g for 10 min. The supernatant was transferred to a fresh tube, and proteins were precipitated with acetone. Acid extracted histone proteins were then collected by centrifugation at 16,000g for 10 min. The supernatant was discarded, and the protein pellet was washed with acetone. The resulting purified histone proteins were resuspended in deionized water and stored at −80°C until processed for Western blotting.

### Western blotting and quantification

The proteins in sample buffer were denatured by keeping at 100°C for 3–5 min in a dry bath. About 1~5ug of histone proteins were separated in a 14% (w/v) SDS-PAGE and transferred onto a nitrocellulose membrane (Bio-rad). After blocking the membrane with 5% (W/V) BSA in Tris-Buffered Saline Tween-20 (TBST), the membrane was incubated overnight with primary antibody, washed in TBST, incubated with Horseradish peroxidase (HRP) conjugated secondary antibody (anti-rabbit IgG, Sigma; anti-mouse IgG, Upstate) at 1:5,000 dilution in TBST, washed and carefully developed with ECL (Pierce). The following primary antibodies were used: H3(ab 1791, Abcam), H3K4me3(39159, Active Motif), H3R2me2(04-808, UBI), H3K27me3 (39156, Active Motif), H3K79me3 (ab 2621, Abcam), H4K20me (ab 9051, Abcam), H3K56ac(07-677, UBI), H3K9me3(39161, Active Motif), H4K20me2(07-367, UBI), H4K16ac(39167, Active Motif), H3K18ac (ab 1191, Abcam), H4R3me2(ab 5823, Abcam), H3K27ac (39685, Active Motif), H3K4me(ab 8895, Abcam), H3K4me2(07-030, UBI). The quality, especially the specificity of all antibodies was first verified experimentally by us (data not shown). The blot was scanned by using Gel Doc XR system (Bio-Rad) with a dynamic range of greater than 3 order of magnitude, and signal intensities were quantified using Image J (http://rsb.info.nih.gov/ij/), a most popular image acquisition tool for accurately calculating area and pixel value, and normalized to values of total H3, which is widely used as a loading control to quantify the amount of modifications of histones.

### Statistical analysis

Statistical analyses were performed using SPSS statistics software. One-way ANOVA was used to evaluate the *p*-value among comparisons, and then followed with a further post-hoc multiple comparisons between groups according to the equal variances assumed or not. Results were presented as group means± SEM. Each group has 3-5 animals. Statistical significance was defined as *p* < 0.05.
